# Asymmetric coevolution of the MEK–ERK binding interface

**DOI:** 10.1016/j.jbc.2025.110708

**Published:** 2025-09-11

**Authors:** Anton V. Persikov, Robert A. Marmion, Stanislav Y. Shvartsman

**Affiliations:** 1 Center for Computational Biology, Flatiron Institute, Simons Foundation, New York, New York, USA; 2 Lewis-Sigler Institute for Integrative Genomics, Princeton University, Princeton, New Jersey, USA; 3 Department of Molecular Biology, Princeton University, Princeton, New Jersey, USA

**Keywords:** protein binding, coevolution, MEK, kinase, short linear motif

## Abstract

The highly conserved extracellular signal–regulated kinase (ERK) regulates diverse cellular processes by phosphorylating a wide range of intracellular substrates. Its catalytic activity relies on phosphorylation by a single upstream kinase, mitogen-activated protein kinase kinase (MEK), which interacts with only a few binding partners. Here, we test whether the asymmetry in protein–protein interaction network architecture influences the coevolution of the MEK–ERK complex. Phylogenetic sequence analysis across metazoan species revealed accelerated divergence in MEK’s intrinsically disordered N-terminal docking motif (docking site [D-site]), whereas ERK remained highly conserved. Structure prediction with AlphaFold2 and extensive molecular dynamics simulations showed that five conserved D-site residues form stable hydrophobic and electrostatic contacts with ERK’s D-recruitment site. Functional assays in *Drosophila melanogaster* confirmed that these D-site interactions are essential for proper downstream signaling and support an allosteric role for this motif. Our results demonstrate that MEK uses a structurally simple yet evolutionarily adaptable motif to regulate MEK–ERK complex stability and binding dynamics. The D-site is strongly conserved within phylogenetic groups such as insects or terrestrial vertebrates, yet diverges across them, reflecting evolutionary pressures that balance functional conservation with signaling adaptability. The presented approach illustrates how the combined approach using sequencing data, molecular simulations, and targeted perturbations can be used to address fundamental questions about the evolution of protein–protein interaction networks.

Protein–protein interactions (PPIs) are central to the regulation of cellular processes, and their evolution reflects the complex interplay between structural, functional, and network-level constraints. A key question is whether proteins with different roles in interaction networks evolve differently. Previous studies found that proteins occupying central positions in PPI networks (so-called hubs) tend to evolve more slowly than their less connected partners, likely because of stronger selective pressures to maintain functional interactions (1, 2). This observation raises the possibility that interacting protein pairs might exhibit asymmetric coevolution, particularly when one protein plays a more central or constrained role in signaling networks.

Several large-scale studies have examined coevolution between protein domains or subunits based on phylogenetic correlations across species. Some focused specifically on domain-level interactions and correlated sequence changes (3, 4). However, these approaches often treat proteins or domains as static units and do not account for the structural details of how specific residues mediate binding. Interpreting coevolving elements benefits from detailed knowledge of domain organization, complementing machine learning–based structural predictions. Despite advances in structural techniques such as cryo-EM, cocrystal structures remain unavailable for many protein complexes. This is especially true for signaling proteins, which often feature intrinsically disordered regions (IDRs) and exhibit dynamic, short-lived interactions.

In this study, we apply a combined approach of bioinformatics analysis, structure prediction, and molecular dynamics (MD) simulations to investigate the mitogen-activated protein kinase kinase (MEK)–extracellular signal–regulated kinase (ERK) complex, a core signaling module in the mitogen-activated protein kinase (MAPK) cascade that regulates essential cellular processes, such as proliferation, differentiation, and apoptosis (5, 6). MEK is the sole kinase that directly activates ERK *via* dual phosphorylation. Activated ERK then phosphorylates a wide variety of downstream effectors, including transcription factors and other kinases, resulting in broad changes in gene expression and cell physiology (7, 8). Dysregulation of MEK, either through mutations or aberrant upstream signaling, can result in inappropriate ERK activation, contributing to pathological conditions, such as cancer and developmental disorders (9, 10). MEK’s substrate specificity and upstream positioning make it a key regulatory node within the MAPK cascade.

A full-length crystal structure of the MEK–ERK complex is not currently available; existing structures are limited to fragments or chimeric peptides of MEK linked to ERK. Most studies primarily focus on the main functional interaction between MEK’s catalytic domain and ERK’s docking groove, where phosphorylation and activation occur (11, 12). The MEK N-terminal docking site (D-site), representing a short linear motif (SLiM) (13), is spatially distinct from the catalytic core, predicted to be intrinsically disordered, and facilitates MEK–ERK binding (Fig. 1*A*). SLiMs are short peptide segments, often located within IDRs, that mediate transient PPIs and evolve rapidly because of structural flexibility and context-dependent binding (14, 15). IDRs are known for their conformational flexibility, allowing them to bind multiple targets and modulate signaling pathways (16, 17). It has been proposed that the D-site could contribute additional regulatory functions, potentially stabilizing the MEK–ERK complex or modulating access to the catalytic site (18).

**Figure 1:**
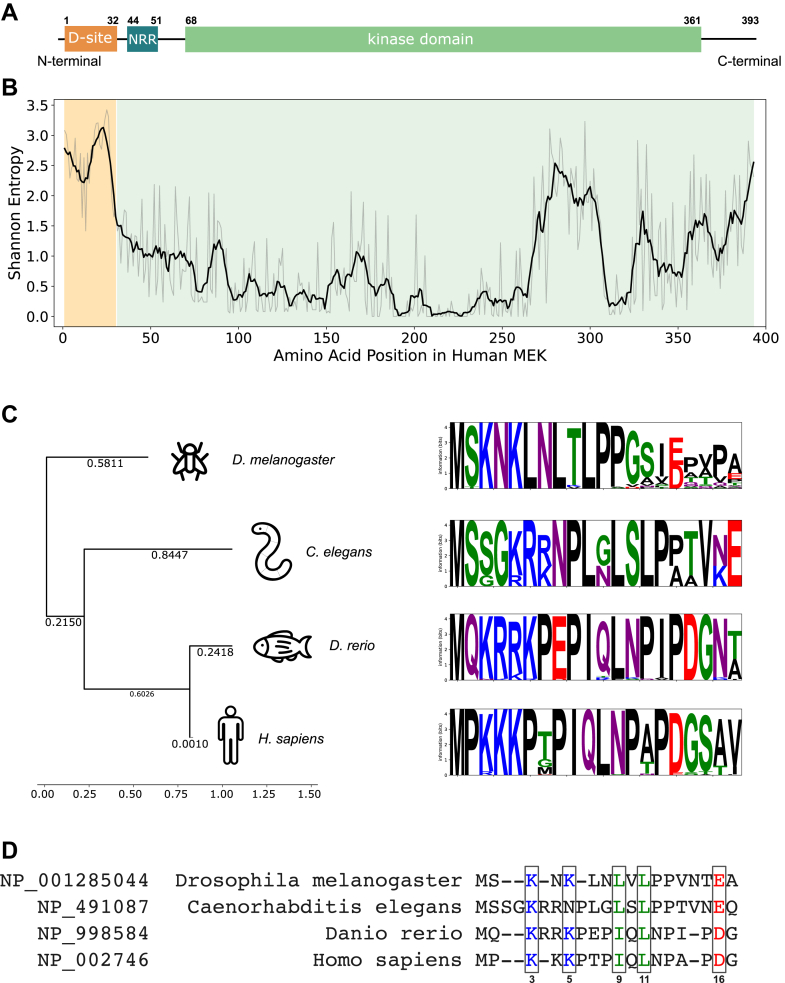
**Bioinformatics analysis of MEK conservation and D-site divergence.***A*, domain organization of MEK, showing the intrinsically disordered D-site near the N terminus. *B*, Shannon entropy showing variability of MEK sequences based on MEK MSA for 309 species. *C*, an evolutionary tree based on the MSA of D-site sequences and four main distinct clusters of conserved D-site sequences illustrated by sequence logos. *D*, best alignment of D-sites across four reference species. Five key D-site positions are framed. The numbers correspond to the sequential number in human MEK. D-site, docking site; MEK, mitogen-activated protein kinase kinase; MSA, multiple sequence alignment.

Initial work by the Nishida group identified the MEK N-terminal D-site as critical for efficient ERK2 binding and activation in cells (19, 20). Later studies confirmed that deletion of the D-site from constitutively active MEK variants abolished their ability to phosphorylate ERK, underscoring its importance (21). Cobb *et al.* (22) expanded on these findings through extensive mutagenesis of MEK1 and ERK2. Mutations in key D-site residues (*e.g.*, K3M, K4M, I9M, L11M) significantly reduced ERK2 binding and phosphorylation, with double and quadruple mutants (K3M/K4M, I9M/L11M, and K3M/K4M/I9M/L11M) showing even stronger effects. These results suggest that residues K3/K4 and I9/L11 in MEK interact with D318/D321 and Y314/Y315 in ERK, although mutating the latter did not impact ERK activity. Further work by Robinson *et al.* (23) identified additional critical ERK residues (K44A and K108A), whose mutation also impaired MEK binding. It was later demonstrated that mutations such as K3M and K6M in the mosquito MEK D-site disrupt ERK phosphorylation, impairing malaria parasite development in *Anopheles gambiae* (24).

Recent advances in structure prediction, such as AlphaFold2, now allow high-confidence modeling of protein complexes, including those involving IDRs (25). Combined with long-timescale MD simulations, this makes it possible to study the conformational behavior of motifs like the D-site and their role in complex stability and binding dynamics.

Here, we integrate coevolutionary analysis with structure prediction and MD simulations to investigate the MEK–ERK interface, with a particular focus on the N-terminal D-site of MEK. Our goal is to understand how conserved and adaptable features coexist within this motif and contribute to binding specificity, regulatory function, and evolutionary divergence.

## Results

### Sequence analysis of the MEK D-site

MEK’s domain organization consists of an N-terminal D-site (residues 1–32), which mediates interactions with downstream ERK, a negative regulatory region (residues 44–51) involved in autoinhibition, and a highly conserved kinase domain (residues 69–361) responsible for ATP binding and substrate phosphorylation (Fig. 1*A*) (26). The D-site is an IDR, which was proposed to allow for transient interactions with ERK (Fig. 1*A*).

To investigate the evolutionary conservation and sequence variability of the D-site, we started with the sequence data from 309 species, originally collected in our previous work (11). The focus of this previous study was on aligning the conserved kinase domain of MEK, whereas the D-site was excluded because of high sequence variability. However, by revisiting these sequences and performing a new multiple sequence alignment (MSA) using the MUSCLE package (27), we identified clear evolutionary patterns.

Utilizing Shannon entropy as the measure of conservation of amino acids in the MEK sequence confirmed that the kinase domain is highly conserved, whereas the D-site shows the highest sequence entropy, reflecting a relaxed evolutionary constraint (Fig. 1*B*). Docking motifs often exhibit this type of variability, as they can accommodate different binding partners while still preserving general function. Despite this variability, the need for MEK to recognize ERK places functional constraints on the D-site sequence, suggesting that its divergence follows distinct evolutionary pressures in different taxa.

Further analysis of the previously excluded D-site sequences identified 201 sequences that clustered into four evolutionary subgroups: terrestrial vertebrates, fish, nematodes, and insects. Within each of these subgroups, sequence conservation was relatively high, as shown by the sequence logos (Fig. 1*C*). Despite significant sequence divergence between groups, conserved sequence motifs were still present, suggesting that the D-site retains key structural and functional elements necessary for ERK docking.

When aligning representative species from each group (*Homo sapiens*, *Danio rerio*, *Caenorhabditis elegans*, and *Drosophila melanogaster*), we identified an updated common pattern in the D-site motif: K/R–X–K/R–X_2-3_–Φ–X–Φ–X_4-5_–D/E, where K/R represents positively charged residues (Lys or Arg), Φ represents hydrophobic residues (usually Leu or Ile), and D/E represents negatively charged residues (Asp or Glu) (Fig. 1*D*). While individual residues varied among groups, this general pattern was maintained, suggesting that the D-site maintains key interactions with ERK even as it diverges between species. The high proline content observed in several sequences also suggests that the D-site remains intrinsically disordered across species, likely enabling its role as a flexible docking module.

Despite its overall sequence divergence, the conservation within subgroups raises an important question: why is the D-site preserved within certain evolutionary lineages, and how does this conservation relate to its functional role in MEK–ERK interactions? The refined sequence alignment provides a foundation for subsequent structural modeling and functional studies, allowing us to assess how sequence divergence translates into differences in docking interactions and regulatory mechanisms across species.

### Structural insights from AlphaFold multimer predictions

**Figure 2:**
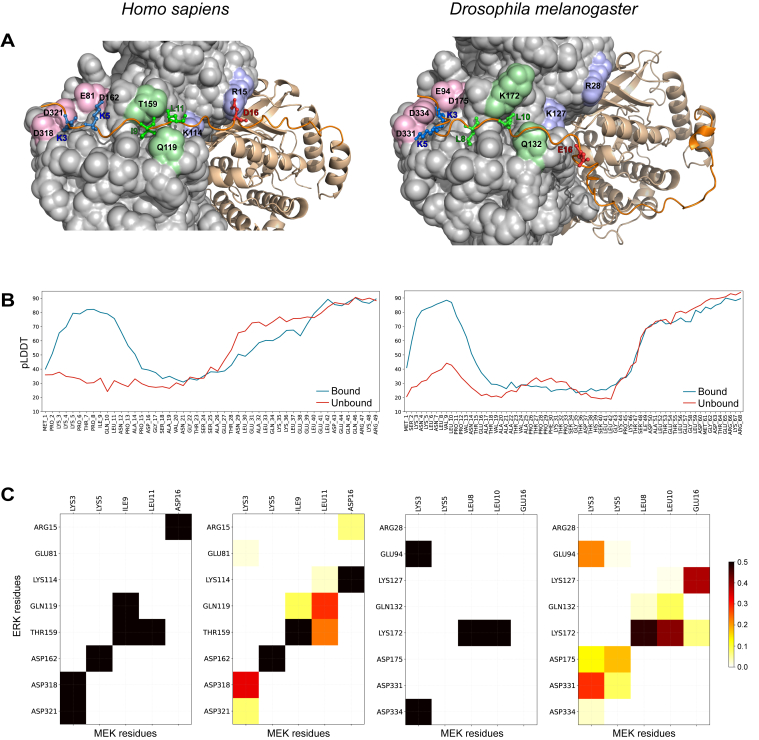
**Structural models and MD-derived contacts defining D-site engagement of the ERK DRS.***A,* structure of the human (*left*) and fly (*right*) MEK–ERK complexes generated by AlphaFold Multimer. The D-site backbone is shown in *dark orange*, and the site chains are shown for the five key amino acids. Contacting MEK residues have *colored labels*, and contacting ERK residues are labeled in *black*. *B*, pLDDT confidence score plot, showing increased ordering of MEK in the complex with ERK (*orange line*) compared with free MEK (*blue line*). *C*, contact maps between D-site key residues and DRS residues of ERK, based on the AlphaFold2 prediction (binary map) and on the contact frequencies observed in 1 μs MD trajectories (color map). DRS, D-recruitment site; ERK, extracellular signal–regulated kinase; MD, molecular dynamics; MEK, mitogen-activated protein kinase kinase; pLDDT, predicted local distance difference test.

To explore how the D-site contributes to MEK–ERK interactions, we used AlphaFold2 Multimer to predict the structure of MEK alone and in complex with ERK for the four representative species identified in our bioinformatics analysis. Despite the sequence and length variability in the N-terminal IDR of MEK, the predicted complexes consistently showed the D-site binding to the same groove in ERK, known as the D-recruitment site (DRS). For comparative analysis, we selected the most evolutionarily distant species among the four references: *H. sapiens* and *D. melanogaster* (Fig. 2*A*). Most of the conserved residues identified through sequence analysis are localized within the DRS of ERK. In the human complex, we identified five key residues (Lys3, Lys5, Ile9, Leu11, and Asp16) that formed electrostatic or hydrophobic pairwise contacts with oppositely charged or hydrophobic residues in the DRS. Fly MEK, which has a longer IDR (Fig. S1), exhibited a difference in D-site interactions, where Glu16 (aligned with human Asp16) did not form any salt bridge in the AlphaFold-predicted structure (Fig. 2*A*).

AlphaFold's predicted local distance difference test (pLDDT) score provides a confidence estimate for atomic positions, with low pLDDT values (<50) indicating intrinsic disorder and high values (>70) corresponding to well-ordered regions. Previous studies demonstrated that pLDDT strongly correlates with intrinsic disorder, making AlphaFold a state-of-the-art tool for identifying disordered regions (25, 28). In both human and fly MEK–ERK complexes, AlphaFold's pLDDT confidence scores indicated that the D-site residues adopted defined conformations upon ERK binding (pLDDT 60–80) compared with their intrinsic disorder in unbound MEK (Fig. 2*B*).

To validate the AlphaFold’s predictions and to further explore conformational flexibility and stability of the interactions, we performed extensive 1 μs MD simulations to assess pairwise amino acid interactions responsible for DRS recognition by the D-site. The MD simulations provided a clearer picture of the interaction between the D-site and ERK, showing that binding is mediated by a combination of electrostatic forces and hydrophobic contacts. As predicted, all five key residues within the D-site, Lys3, Lys5, Ile9, Leu11, and Asp16, were critical for stabilizing the binding interface.

Contact maps comparing AlphaFold predictions and MD-derived contact occupancies for the human and fly MEK–ERK complexes are shown in Figure 2*C*. While AlphaFold predicted different static conformations for the two species, MD simulations revealed common interaction patterns. The simulations identified multiple binding modes and key amino acid interactions within the D-site, confirming the importance of previously proposed residues, such as Lys5, which formed a stable salt bridge with Asp162 of ERK. In addition, we identified new interactions that had not been previously characterized. In particular, Lys3 interacted with Glu81 and Asp318/Asp321, whereas hydrophobic residues, Ile9 and Leu11, remained in close contact with Gln119 and Thr159. Interestingly, Asp16, which AlphaFold positioned near Arg15, instead formed a more frequent salt bridge with Lys114 in MD simulations, a feature observed in both human and fly complexes.

The structural adaptability of the D-site, accommodating species-specific sequence differences while maintaining core interactions, aligns with its evolutionary conservation within clusters. These findings support the hypothesis that five conserved residues are essential for ERK binding and for maintaining MEK's functional activity.

### Functional residue identification by point mutation simulations

Having identified the key residues in the D-site, we sought to perturb them and assess their impact on MEK–ERK binding. First, we individually mutated each key residue to alanine and performed AlphaFold predictions of the resulting MEK–ERK complexes. In all single-point mutations, the D-site remained bound to the ERK docking groove. Only when all five residues, K3A, K5A, I9A, L11A, and D16A, were mutated simultaneously (MEK^5A^ mutant), the D-site no longer contacted the DRS groove in the AlphaFold prediction.

**Figure 3:**
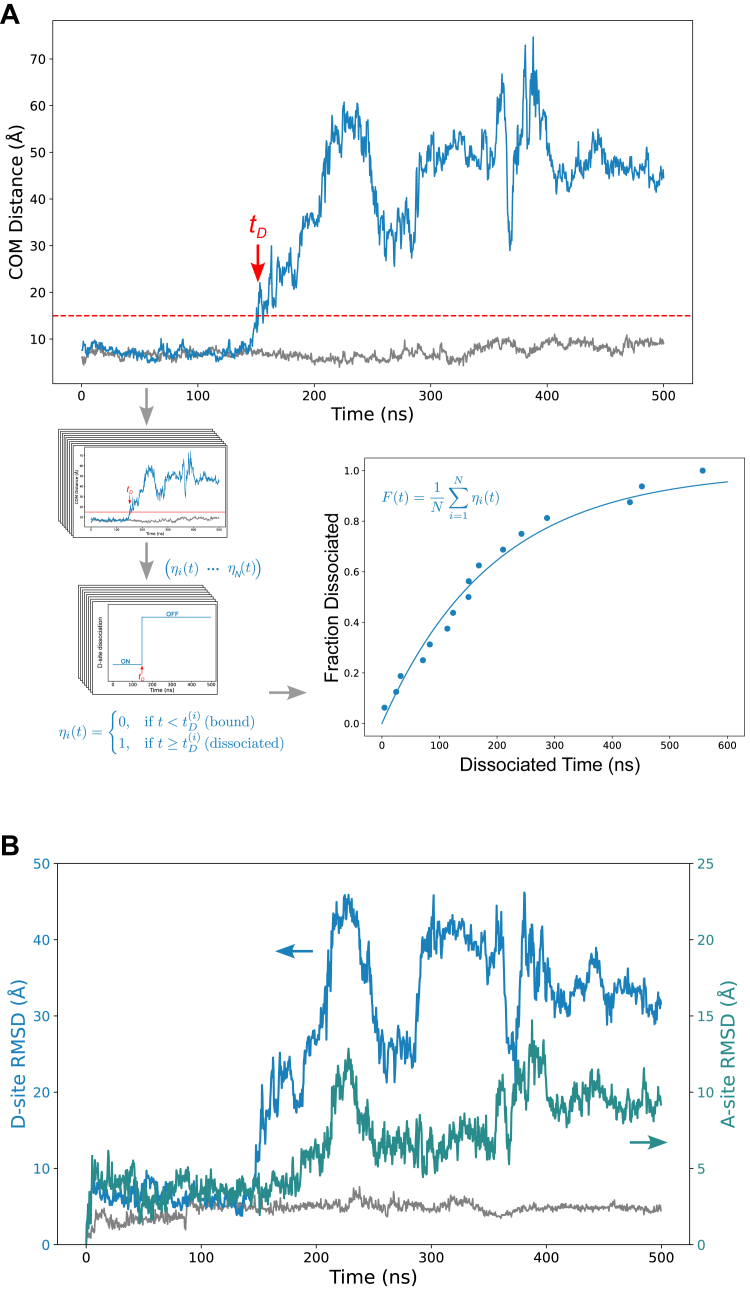
**MD simulations reveal allosteric coupling in MEK–ERK interaction.***A*, COM distance between MEK D-site and ERK DRS during a sample MD simulation for the MEK^5A^ mutant–ERK complex (*blue line*), compared with the WT MEK–ERK complex (*gray line*). The fraction-dissociated curve indicates the cumulative fraction of trajectories (total n = 16) exceeding the 15 Å COM dissociation threshold within the simulation time. *B*, RMSD plots comparing the flexibility of the D-site (*blue line*, *left axis*) with the MEK’s active site (*green line*, *right axis*). The RMSD for the WT MEK–ERK complex is shown for the reference (*gray line*). Both the D-site and A-site exhibit increased flexibility after D-site attachment (around the 150 ns time point). COM, center of mass; D-site, docking site; DRS, D-recruitment site; ERK, extracellular signal–regulated kinase; MD, molecular dynamics; MEK, mitogen-activated protein kinase kinase.

Following these structural predictions, we performed 1 μs MD simulations for both WT MEK and MEK^5A^, starting from the AlphaFold-predicted conformation where the D-site was initially attached to ERK (as in Fig. 2*A*), but with the five alanine substitutions introduced. To monitor conformational changes, we measured the center of mass (COM) distance between the five key D-site residues in MEK and eight residues in the ERK DRS (as listed in Fig. 2*C*). In the WT simulation, the D-site remained bound throughout 1 μs, with the COM distance fluctuating between 5 and 10 Å (Fig. 3). In contrast, in the MEK^5A^ mutant, the D-site detached from the ERK groove, with the COM distance increasing dramatically. Figure 3*A* shows the computationally predicted kinetics of the D-site detachment, defined as the moment when the COM distance exceeded an arbitrary threshold of 15 Å. Across all simulations, detachment occurred within the first 600 ns, as shown in the cumulative fraction–dissociated curve. Further MD simulations on quadruple alanine mutants (MEK^4A^-K3, MEK^4A^-K5, MEK^4A^-I9, MEK^4A^-L11, and MEK^4A^-D16), each retaining only one original key residue, showed no stable dissociation during 1 μs, confirming that each residue significantly contributes to the stability of the MEK–ERK interaction (Fig. S3).

To further characterize the structural changes, we used RMSD to track the stability and mobility of different regions of MEK over time. As expected, RMSD analysis showed a correlated increase in structural deviations at the D-site detachment, as shown in Figure 3*B*, for the same MD trajectory from Figure 3*A*. More strikingly, D-site detachment also led to increased flexibility of the MEK active site (residues 216–222), despite the rest of MEK remaining bound to ERK. The loss of D-site interactions resulted in a significant rise in active site fluctuations, suggesting that the D-site plays a role in stabilizing the active conformation of MEK. Given that kinase activity depends on maintaining precise positioning of catalytic residues, this increased mobility likely has functional consequences for ERK activation, similar to the induced fit mechanism observed in other kinase-substrate systems. These findings support the hypothesis that the D-site is not only essential for ERK binding but also plays a structural role in regulating MEK’s active site stability, with potential implications for its catalytic function.

### Functional assessment of the MEK D-site *in vivo*

**Figure 4:**
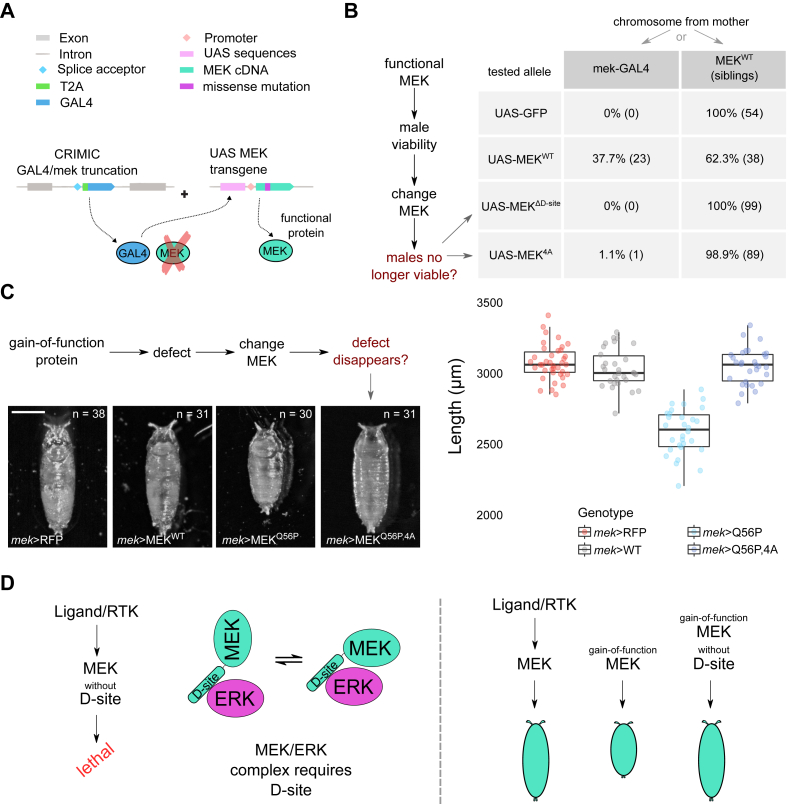
**In vivo assays demonstrate essential MEK D-site function for ERK signaling and viability.***A*, a schematic of the CRIMIC split-expression system used to test MEK function in *Drosophila*. The endogenous *mek* gene, which is located on the X chromosome, is replaced by a T2A-GAL4 cassette, eliminating all native MEK protein and providing GAL4 expression under *mek* regulatory sequences. Functional MEK is supplied by a UAS-MEK transgene (WT or mutant), whose expression is specifically activated by the *mek*-GAL4 cassette. *B*, genetic strategy and scoring: In each cross, half of the male progeny inherit the *mek*-GAL4 (*mek*-null) X chromosome and require the UAS-MEK transgene for survival, whereas the other half inherit a WT *mek* X chromosome from the mother and develop normally as internal controls ("siblings"). The table shows survival rates for tested alleles and siblings, highlighting that only WT MEK rescues male viability. MEK mutants lacking the D-site (ΔD-site or 4A) fail to rescue. *C*, representative pupal images for each genotype. Constitutively active MEK (Q56P) produces a gain-of-function phenotype, shortened pupal length, that is suppressed when D-site mutations are combined with Q56P (Q56P,4A). Quantitative analysis of pupal length for each genotype (n shown in the *panel*), presented as box-and-whisker plots. *D*, summary models: *Left,* the D-site on MEK is essential for MEK–ERK complex formation and viability. *Right,* D-site mutations eliminate both normal and gain-of-function phenotypes, confirming that MEK–ERK docking *via* the D-site is required in both physiological and pathological contexts. D-site, docking site; ERK, extracellular signal–regulated kinase; MEK, mitogen-activated protein kinase kinase.

To evaluate the functional relevance of the MEK D-site in a developmental context, we used a previously developed *mek* loss-of-function allele in *D. melanogaster*, in which the endogenous *mek* coding sequence, located on the X chromosome, is replaced by a *mek*-GAL4 cassette (Fig. 4*A*) (29). This CRIMIC split-expression system physically separates the *mek* genomic locus from any functional MEK protein, ensuring that the animal’s only source of MEK comes from a rescuing UAS-MEK transgene. Because *mek* is X-linked, *Drosophila* males (who possess only one X chromosome) are especially sensitive to MEK loss, serving as an unambiguous and stringent readout for the functional rescue provided by each transgenic construct. In this context, male survival is a direct proxy for MEK-dependent ERK activation: only males that receive a functional MEK transgene survive to adulthood.

Earlier studies in cultured cells demonstrated that the MEK D-site is critical for ERK signaling, but these experiments could not resolve whether the D-site is absolutely essential for all signaling or only a substantial portion because of confounding effects of overexpression (21, 22, 30). The *Drosophila* split-expression system overcomes this limitation, providing a simple, physiologically relevant setting to test whether the D-site is required for MEK function at endogenous levels across diverse tissues during development. Here, we ask directly whether D-site mutant MEK transgenes can rescue male viability in the complete absence of endogenous MEK.

To rigorously test this, we used a straightforward genetic cross (Fig. S4). Since *mek* is X-linked, *mek*-GAL4/+ females crossed to UAS-MEK males yield approximately half their male progeny with a WT X chromosome (serving as internal controls or "siblings") and half with the *mek*-GAL4 null allele (the "test" cohort). The siblings and tested males can be easily distinguished by visible genetic markers, such as balancer chromosomes carrying a dominant eye marker, ensuring clear genotype assignment for each animal. Only males carrying the *mek*-GAL4 (MEK null) X chromosome are dependent on the rescuing transgene; their siblings, inheriting the WT *mek* allele, develop normally and provide a robust internal control for overall genetic background and viability.

As a negative control, we validated that transgenes lacking MEK function, such as UAS-GFP, are unable to rescue male viability (Fig. 4*B*). In contrast, the WT UAS-MEK transgene fully restores male survival to rates comparable with WT siblings, confirming that our system faithfully reads out MEK function *in vivo*. We next tested a truncated version of MEK lacking the N-terminal 27 amino acids (MEK_ΔD-site), which completely removes the D-site region. In these rescue experiments, MEK_ΔD-site failed to support male viability, demonstrating that the D-site is strictly required for MEK-dependent ERK activation and normal development. To interrogate the contribution of specific residues within the D-site, we generated a quadruple alanine substitution mutant (MEK^4A^: K3A, K5A, I9A, and L11A), targeting residues predicted to make direct contacts with ERK’s DRS. Like the deletion mutant, MEK^4A^ failed to rescue *mek*-null males, reinforcing that side-chain interactions involving Lys3, Lys5, Ile9, and Leu11 are indispensable for MEK–ERK complex formation and productive signaling *in vivo*. The inability of these mutants to rescue viability cannot be attributed to transgene expression, genetic background, or general toxicity, as WT siblings develop normally in every cross.

Beyond assessing loss of function, we also tested whether D-site mutations can suppress the effects of a constitutively active MEK allele, MEK_Q56P (11, 31). This mutation generates MEK proteins that are catalytically active without upstream phosphorylation but whose kinetics remain similar to fully activated WT MEK (32, 33). When MEK_Q56P is expressed at physiological levels under *mek*-GAL4 control, it produces a reproducible gain-of-function phenotype: pupae exhibit a noticeably shortened anterior–posterior axis (Fig. 4*C*), consistent with increased ERK pathway activation. While the precise mechanism remains unclear, this phenotype likely reflects disruption of multiple ERK-dependent developmental processes, including altered timing of pupation, growth regulation by the prothoracic gland, or abnormal systemic metabolism (34, 35, 36, 37). Importantly, introducing the MEK^4A^ D-site mutations in *cis* with Q56P abolishes the shortened pupal phenotype: animals are indistinguishable from WT controls, indicating that even constitutively active MEK strictly depends on D-site–DRS interactions to signal through ERK.

Together, these results demonstrate that the MEK D-site is absolutely required for ERK activation and normal development *in vivo* and that its disruption eliminates both normal and pathological MEK function. The system described here, leveraging X-linked genetics, visible markers, and direct viability assays, provides a powerful and specific means to dissect MEK–ERK interactions in the context of the whole organism, supporting computational and biochemical predictions that Lys3, Lys5, Ile9, and Leu11 are critical for stable and functional MEK–ERK complex formation.

## Discussion

The emergence of tools such as AlphaFold2 has enabled confident predictions of protein complex structures, including transient signaling assemblies like MEK–ERK. In parallel, improvements in MD algorithms and access to high-performance computing now allow simulations of protein complexes on the microsecond timescale in explicit solvent environments, modeling systems with hundreds of thousands of atoms. Leveraging these capabilities, we performed structure prediction and extensive MD simulations to investigate the MEK–ERK complex, specifically focusing on the interactions between MEK’s D-site and ERK’s DRS groove, as well as the conformation and dynamics of the active site.

Our study revealed that while ERK’s side of the binding interface is highly conserved across more than 300 species, MEK shows markedly lower conservation in its D-site, despite similar overall evolutionary constraints. By focusing on the N-terminal region of MEK, which includes the D-site and was previously omitted from large-scale analyses (11), we identified four major sequence groups, each showing strong intragroup conservation: terrestrial vertebrates (from frog to human), fish, nematodes, and insects (Fig. 1*B*). This divergence in coevolutionary trajectories of ERK and MEK, particularly centered on the DRS–D-site interface, highlights a striking example of asymmetric coevolution (1). Structural predictions and MD simulations confirmed that the D-site consistently engages the DRS across species, suggesting functional constraints on this interaction despite sequence divergence. This evolutionary asymmetry may reflect MEK’s need to adapt its docking motif under varying physiological contexts while maintaining effective ERK binding.

The MEK D-site conforms to a classical SLiM, aligning with broader patterns observed in motif evolution. SLiMs often rapidly diverge because of intrinsic flexibility, yet retain key physicochemical features required for binding (14, 15). Our network-centric analysis complements this structural framework by highlighting asymmetric coevolution driven by functional constraints.

Our integrative approach, combining sequence analysis, structural prediction, and dynamic simulations, refined the understanding of key residues mediating the MEK D-site–ERK DRS interaction: some previously known, and others newly revealed, such as MEK Asp16. Our data also captured multiple conformational states of the MEK–ERK complex, suggesting that the DRS–D-site interface is dynamic and contributes to binding adaptability. Despite evolutionary divergence, interaction maps from human and *Drosophila* complexes showed a similar pattern of hydrophobic contacts and salt bridges, pointing to a conserved mode of molecular recognition shaped by both structural constraints and evolutionary tuning.

Despite the observed striking asymmetry in MEK–ERK coevolution, the D-site is highly conserved within species groups, including terrestrial vertebrates, suggesting functional constraints. Notably, missense mutations at positions identified in our study are extremely rare in clinical databases. In fact, no variation in key residues involved in MEK–ERK interaction was found in large-scale human variant datasets, including the UK Biobank, which comprises over 500,000 sequenced healthy individuals. These observations are consistent with deep mutational scanning studies of human ERK, which identified the ERK DRS residues highlighted here as among the most deleterious when mutated, including Glu81, previously overlooked but ranked as the most deleterious (38). Together, these findings suggest that although the D-site evolves flexibly across species, it is under strong purifying selection in humans, likely because of its crucial role in maintaining proper MEK–ERK docking and downstream signaling fidelity. Thus, evolutionary plasticity in the D-site sequence is balanced by stringent structural and functional constraints, reconciling observed cross-species flexibility with strong within-group conservation.

Although speculative, variation in MEK linker length across species may modulate D-site accessibility and ERK binding dynamics; this hypothesis merits future investigation. In *Drosophila*, the linker is extended by 19 residues, possibly enabling flexible tethering and dynamic exchange, whereas shorter linkers in vertebrates may allow MEK to serve as a more persistent ERK anchor, influencing phosphorylation efficiency. Given that ERK functions as a central hub interacting with numerous partners, competition at its D-site is critical (1). Beyond MEK, the ERK DRS accommodates diverse partners with their own docking motifs, including substrates (RSK1, MNK1, MSK1, and caspase-9), phosphatases (DUSP6 and HePTP), scaffolds (PEA-15), and negative regulators (LRRC4) (39). Transcription factors, such as Elk-1, also rely on DRS binding for activation and gene regulation (40). In *Drosophila*, the transcriptional repressor capicua (Cic) engages ERK *via* a reverse D-site to regulate localization and signaling specificity (41). These overlapping docking specificities suggest that linker length may fine-tune MEK–ERK binding kinetics and substrate competition at the DRS. Future studies could design MEK variants with modified or swapped linkers to investigate how these differences impact ERK binding, phosphorylation kinetics, and broader MAPK signaling dynamics. It will also be interesting to explore whether the multisite binding and asymmetric coevolution observed in MEK–ERK interactions extend to other MAPK signaling modules.

In addition to refining the direct MEK–ERK binding interface, our results suggest a potential allosteric mechanism of regulation. Although the MEK active site and ERK phosphorylation site are spatially distant from the D-site–DRS interface, MD simulations revealed increased mobility of MEK’s active site upon D-site detachment (see RMSD plot in Fig. 3), indicating a possible allosteric coupling. This long-range effect implies that loss of D-site engagement may destabilize the active site, modulating MEK’s catalytic function. Similar allosteric mechanisms have been observed in other MAPK pathways and may represent a conserved regulatory feature of the MEK–ERK signaling module. Supporting this, *Drosophila* experiments demonstrated the functional importance of the D-site, as MEK variants lacking it failed to rescue male viability (Fig. 4). The observed coupling echoes regulatory strategies in the p38 MAPK pathway, which shares structural and mechanistic features with ERK signaling (42). In that system, p38 interacts with upstream kinases such as MKK6 *via* D-site/DRS-like docking, and an N-terminal extension in MKK6 plays a critical role in modulating binding and activation dynamics. Structural studies show that this docking enhances p38 activity both through allosteric modulation and an anchor effect (43). Applying our integrative approach to other MAPK modules, such as MKK–p38, could uncover conserved principles of allosteric regulation across the MAPK family.

## Experimental procedures

### Multiple sequence alignments

To perform MSA for MEK1 proteins, we collected 309 orthologous sequences from metazoan species using the protein IDs identified in our previous study (dataset S2 from Ref. (11)). The *H. sapiens* MEK1 sequence (A4QPA9) was used as the reference. MSA was carried out using the MUSCLE v.5.3 (27). In contrast to our prior study, which focused exclusively on the conserved kinase domain and thus excluded the variable N-terminal region, here, we specifically analyzed the MEK D-site within this previously excluded region. To this end, we extracted the N-terminal regions from the 309 MEK sequences. Sequences lacking these regions were excluded, resulting in a final set of 274 sequences. These were then aligned using MUSCLE with increased gap open and gap extension penalties (gap open: 3.0, gap extend: 0.5) to improve alignment of the short and variable D-site region.

To cluster D-site sequences, we constructed a sequence similarity network using the TreeConstruction module from Biopython. Two sequences were connected if their pairwise sequence distance was below 0.2 (*i.e.*, ≥80% identity). The resulting network was clustered using the K-means clustering algorithm, revealing four distinct sequence groups.

For ERK2, we used the *H. sapiens* ERK2 sequence (P28482) as a query in a BLASTp search against the National Center for Biotechnology Information nonredundant protein database to identify closely related orthologs. Partial and redundant entries were removed, and the top 300 nonredundant full-length sequences were aligned using MUSCLE.

Representative MEK and ERK sequences from four reference species (*H. sapiens*, *D. rerio*, *C. elegans*, and *D. melanogaster*) were aligned separately using MUSCLE (Figs. S1 and S2). The phylogenetic tree (Fig. 1*C*) was generated from the full-length MEK sequences and visualized using Molecular Evolutionary Genetics Analysis software (Pennsylvania State University) (44).

Shannon entropy was computed from MSA data to quantify sequence variability at each alignment position (lower entropy indicates greater conservation) (45). Sequence logos were created using the Python package Logomaker (46).

### Protein structure prediction using AlphaFold2

To model MEK–ERK complexes across selected species, we used AlphaFold2 Multimer (25) with default multimer settings and full database search mode. The following UniProt IDs were used: human MEK (A4QPA9), human ERK (P28482), fly MEK (Dsor1) (Q24324), and fly ERK (rl) (P40417). Protein sequences were formatted in FASTA files as input. The prediction process was separated into two stages: feature generation and structure folding. Each stage was run using custom wrapper scripts and submitted as batch jobs to a high-performance computing cluster.

For each complex, 25 AlphaFold2 models were generated, exhibiting minimal structural variation in the D-site–DRS interface, from which the top-ranked model was selected for further MD analyses. Model confidence and structural features were analyzed based on AlphaFold’s output, including the pLDDT. Structural models were visualized using ChimeraX, v1.17 (University of California, San Francisco (UCSF)). Amino acid interaction maps were generated using a custom Python script with Biopython (47), based on interchain residue pairs with any nonhydrogen atom within 4 Å.

### MD simulations

Production MD simulations were performed in the NPT ensemble for 1 μs (five replicates for WT MEK–ERK complexes; 16 replicates at 375 K for the MEK^5A^ mutant), using OpenMM’s CUDA implementation (48). Before production runs, systems underwent 1000 steps of energy minimization and equilibration using a Langevin integrator (4 fs timestep with hydrogen mass repartitioning), the CHARMM36 force field (49), rigid water, hydrogen bond constraints, and barostatic pressure control (applied every 25 steps). The starting structure was obtained from the AlphaFold2 prediction (ranked_0.pdb model) and subsequently prepared for simulation using CHARMM-GUI (50, 51). The system was solvated in a rectangular TIP3P water box with a 10 Å padding distance from the solute and ionized with 0.15 M KCl using Monte Carlo ion placement to neutralize the net charge. The final solvated periodic box system measured approximately 113 Å per side. Long-range electrostatic interactions were treated using the particle mesh Ewald method, with the grid generated by fast Fourier transforms. The system was initially equilibrated under NVT conditions (constant volume and temperature) at 300 K. Production MD simulations were then carried out in the NPT ensemble (constant pressure and temperature) for 1 μs, using OpenMM’s CUDA implementation on a GPU-accelerated platform (48). Standard settings were used for bond constraints and integration time steps, with periodic boundary conditions applied throughout the simulation.

### Analysis of D-site dissociation from MD simulations

To identify D-site dissociation events, the COMs were calculated using backbone atoms of key residues: MEK D-site (3, 5, 9, 11, 16) and ERK DRS (15, 81, 114, 119, 159, 162, 318, 321). For each MD simulation trajectory *i* = 1,2, … ,*N*, the COM distance was calculated as a function of time. The dissociation time *t*_*D*_^*(i)*^ for trajectory *i* was defined as the earliest time point at which the COM distance exceeded a threshold of 15 Å.

For each trajectory *i*, a binary time-dependent indicator function *η*_*i*_*(t)* was constructed to represent the dissociation state:

$$\eta_{i}\left(t\right)=\left\{ \begin{array}{l} 0,t<t_{D}^{\left(i\right)}\;\left(bound\right)\\ 1,t\geq t_{D}^{\left(i\right)}\;\left(dissociated\right) \end{array}\right.$$

The cumulative fraction of dissociated trajectories at time *t* was computed by averaging *η*_*i*_*(t)* over all *N* trajectories:

$$F\left(t\right)=\frac{1}{N}\sum_{i=1}^{N}\eta_{i}\left(t\right)$$

The function *F(t)* increases monotonically from 0 to 1 (or less than 1 if some trajectories did not dissociate).

The RMSD of the given subdomain (*e.g*., D-site or A-site) relative to its initial conformation was calculated as a function of time:

$${RMSD}_{i}\left(t\right)=\sqrt{\frac{1}{M}\sum_{m=1}^{M}{\left\|r_{m}\left(t\right)-r_{m}\left(0\right)\right\|}^{2}}$$

where *r*_*m*_*(t)* is the position of atom *m* at time *t*, and *M* is the total number of atoms in the subdomain.

### Fly strains and rearing

The T2A-GAL4 line of *mek* (fly *Dsor1*) was previously generated (29) (BDSC #78914). A previously generated stock of UAS-Dsor1 was used for rescue experiments (33). Additional UAS lines were 10XUAS-IVS-mCD8::RFP (BDSC #32219), UAS-mCD8::GFP (BDSC #5137), and MEK_Q56P (11). All fly stocks were reared under standard laboratory conditions (cornmeal food in vials stored at 25 °C).

### Generation of additional MEK alleles

D-site deletions/substitutions were introduced into UAS-MEK_Q56P and UAS_MEK constructs with site-directed mutagenesis using Q5 hot start DNA Polymerase (NEB #M0493) with mutagenic primers. Gibson assembly of final vectors was accomplished with HiFi Assembly Master Mix (NEB #E2621S) into vector pTIGER digested with NheI and XbaI. Constructs were integrated into the attP40 site using the ΦC31-based integration system (52, 53, 54).

**Table:** 

Primers	
mutate_alanines_R	cagggcgttggcCGACATCTGATAATAATTAATTAAGAC
del_2to47_F	AGCATCGATGCGCTG
del_2to47_R	CATCTGATAATAATTAATTAAGACGTCAG
pTIGER2mekmutA_F	cagagaggccggcagagaggctagcATGTCGGCCAACGCCCTG
pTIGER2mekmutA_R	gttaacgttcgaggtcgactctagaTTAGTTGGGCGACGTATTACGC
pTIGER2mekdel47_F	cagagaggccggcagagaggctagcATGAGCATCGATGCGCTG
pTIGER2mekdel47_R	gttaacgttcgaggtcgactctagaTTAGTTGGGCGACGTATTAC

## Data availability

The coordinate file for the AlphaFold2-predicted *H. sapiens* MEK–ERK complex has been deposited in Zenodo under DOI 10.5281/zenodo.16808770, and the corresponding *D. melanogaster* MEK–ERK complex is available under DOI 10.5281/zenodo.16808787. All other data supporting the findings of this study are contained within the article and its supporting information.

## Supporting information

This article contains supporting information.

## Conflict of interest

The authors declare that they have no conflicts of interest with the contents of this article.
