# Supplementary material for: Asymmetric coevolution of the MEK–ERK binding interface
Source: J Biol Chem. 2025 Sep 11;301(10):110708. doi: 10.1016/j.jbc.2025.110708 (PMC12550783; doi:10.1016/j.jbc.2025.110708)
Supplement: Figure S1 [file mmc1.pdf]

```
A4QPA9_Hsap      MP--KKK-PTPIQLNPAP-DGSAVNG--------------------TSSA--ETNLEALQK
Q4V947_Drer      MQ-KRRK-PEPIQLNPIP-DGNAING--------------------TGAT--ETNLEALQK
Q10664_Cele      MSSGKRRNPLGLSLPPTVNEQSES-G--------------------EATAEEATATVPLEE
Q24324_Dmel      MS--KNK--LNLVLPPVNTEATVAAATVAPTPPFKTPSGTDTHSLLGKP--KTSIDALTE
                 *.  ::: :  : *.*.  :..  .:                    . .  .*...:*.:

A4QPA9_Hsap      KLEELELDEQQRKRLEAFLTQKQKVGELKDDDFEKISELGAGNGGVVFKVSHKPSGLVMA
Q4V947_Drer      KLEELELDEQQKKRLEAFLTQKQKVGELKDDDFEKISELGAGNGGVVFKVLHRPSGFIMA
Q10664_Cele      QLKKLGLTEPQTQRLSEFLQVKEGIKELSEDMLQTEGELGHGNGGVVNKCVHRKTGVIMA
Q24324_Dmel      TLEGLDMGDTERKRIKMFLSQKEKIGELSDEDLEKLGELGSGNGGVVMKVRHTHTHLIMA
                 .*:.*.: : :.:*:. **.:*::::**::::::: :***.****** *: *. ::.:**

A4QPA9_Hsap      RKLIHLEIKPAIRNQIIRELQVLHECNSPYIVGFYGAFYSDGEISICMEHMDGGSLDQVL
Q4V947_Drer      RKLIHLEIKPAIRNQIIRELQVLHECNSPYIVGFYGAFYSDGEISICMENMDGGSLDQCL
Q10664_Cele      RKLVHLEIKPSVRQQIVKELAVLHKCNSPFIVGFYGAFVDNNDISICMEYMDGLSLDIVL
Q24324_Dmel      RKLIHLEVKPAIKKQILRELKVLHECNFPHIVGFYGAFYSDGEISICMEYMDGGSLDLIL
                 ***:***:**:::.**::**.***:**.*:********::::******.***.*** .*

A4QPA9_Hsap      KKAGRIPEQILGKVSIAVIKGLTYLREKHKIMHRDVKPSNILVNSRGEIKLCDFGVSGQL
Q4V947_Drer      KKAGKIPEQILGKVSIAVIKGLSYLREKHKIMHRDVKPSNILVNSRGEIKLCDFGVSGQL
Q10664_Cele      KKVGRLPEKFVGRISVAVVRGLTYLKDEIKILHRDVKPSNMLVNSNGEIKLCDFGVSGML
Q24324_Dmel      KRAGRIPESILGRITLAVLKGLSYLRDNHAIIHRDVKPSNILVNSSGEIKICDFGVSGQL
                 *::*::**.::*::::**::**:**::.::*:********:****.****:*******:*

A4QPA9_Hsap      IDSMANSFVGTRSYMSPERLQGTHYSVQSDIWSMGLSLVEMAVGRYPIPPPDAKELELMF
Q4V947_Drer      IDSMANSFVGTRSYMSPERLQGTHYSVQPDIWSMGLSLVEMAIGRFPIPPPDAKELEQIF
Q10664_Cele      IDSMANSFVGTRSYMAPERLTGSHYTISSDIWSFGLSLVELLIGRYPVPAPSQAEYATMF
Q24324_Dmel      IDSMANSFVGTRSYMSPERLQGTHYSVQSDIWSLGLSLVEMAIGMYPIPPPNTATLESIF
                 ***************:****:*:**:::****:******::*::*:*:*:..::: :*

A4QPA9_Hsap      GCQVEGD--AAETP--PRPRTPGRPLSSYGMDSRPPMAIFELLDYIVNEPPPKLPSGVFS
Q4V947_Drer      GQPLEGDPSASDTS--PKPRPPGRPGSSYGPDSRPPMAIFELLDYIVNEPPPKLPS-IFG
Q10664_Cele      NVA-ENEIELADSLEEPNYHPPSNP---------ASMAIFEMLDYIVNGPPPTLPKRFFT
Q24324_Dmel      ADNAEES--G---------QPTDEP---------RAMAIFELLDYIVNEPPPKLEHKIFS
                 .   *.:   .:.   :...::..* .... ... .*****:******:***:*:. :*.

A4QPA9_Hsap      LEFQDFVNKCLIKNPAERADLKQLMVHAFIKR-SDA-EEVDFAGWLCSTIGLNQPSTPTH
Q4V947_Drer      AEFQDFVNKCLIKNPAERADLKQLMVHSFIKD-SEA-EEVDFAGWLCSTIGLNQPATPTH
Q10664_Cele      DEVIGFVSKCLRKLPSERATLKSLTADVFFTQYADHDDQGEFAVFVKGTINLP-KLNP--
Q24324_Dmel      TEFKDFVDICLKKQPDERADLKTLLSHPWIRK-AEL-EEVDISGWVCKTMDLP-PSTPKR
                  *: :**:.** * *.***:**.*..: ::.. ::  ::.:::::::.*:.*..:.:*..

A4QPA9_Hsap      -AAGV
Q4V947_Drer      -SVGM
Q10664_Cele      -----
Q24324_Dmel      NTSPN
                  . .
```

Supplementary Figure 1.
Multiple sequence alignment of MEK’s four reference species: Homo sapiens, Danio rerio, Caenorhabditis elegans, and Drosophila melanogaster.
