# Supplementary material for: Asymmetric coevolution of the MEK–ERK binding interface
Source: J Biol Chem. 2025 Sep 11;301(10):110708. doi: 10.1016/j.jbc.2025.110708 (PMC12550783; doi:10.1016/j.jbc.2025.110708)
Supplement: Figure S3 [file mmc3.pdf]

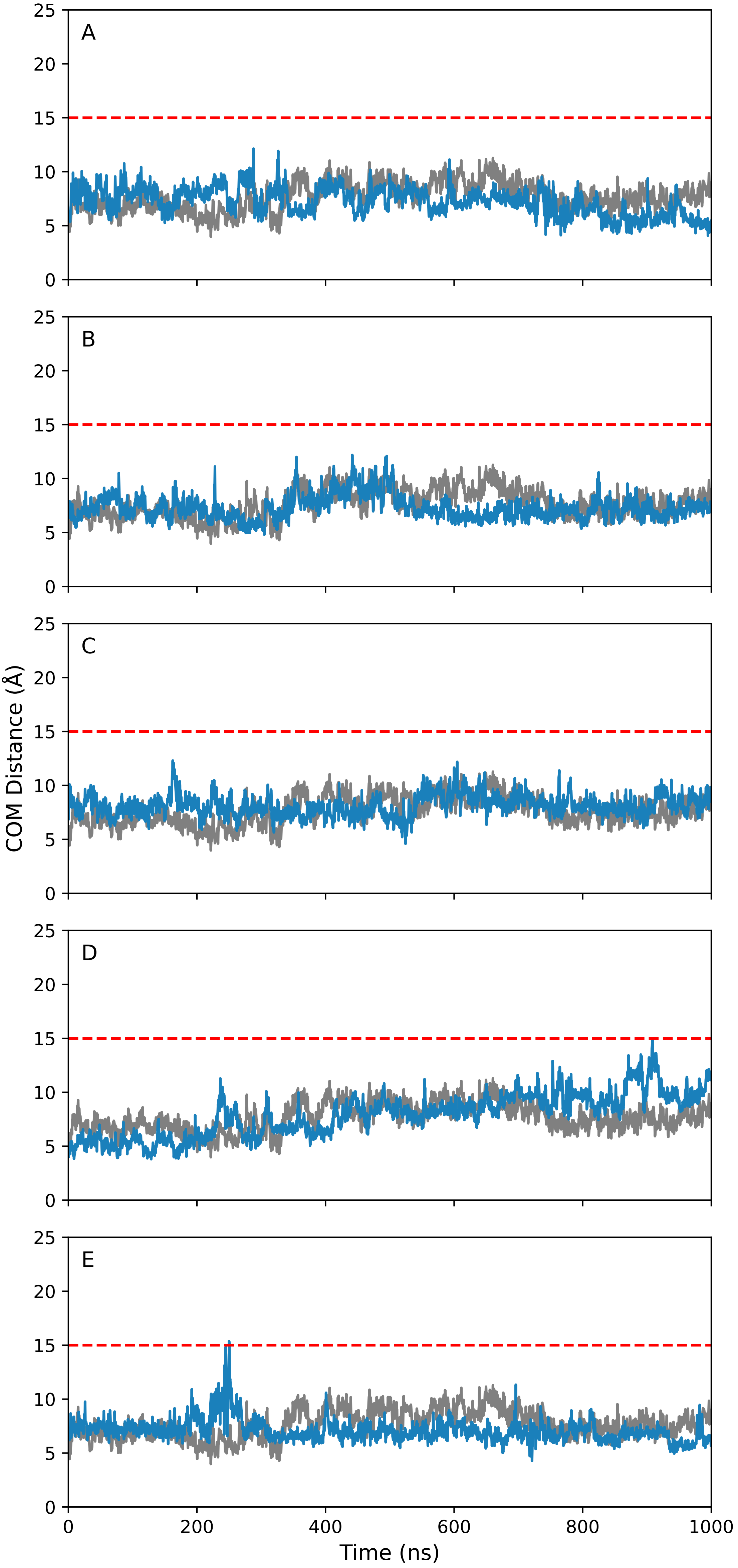

Supplementary Figure S3.
MD simulations of quadruple alanine mutants retaining only one original D-site residue. Center-of-mass (COM) distances between the MEK D-site and ERK DRS are plotted over 1 µs for each quadruple mutant: (A) MEK^{4A}-K3, (B) MEK^{4A}-K5, (C) MEK^{4A}-I9, (D) MEK^{4A}-L11, and (E) MEK^{4A}-D16. No stable dissociation was observed for any mutant within this timeframe; the dashed horizontal line indicates the 15Å dissociation threshold used in Figure 3A.
