# Supplementary material for: Asymmetric coevolution of the MEK–ERK binding interface
Source: J Biol Chem. 2025 Sep 11;301(10):110708. doi: 10.1016/j.jbc.2025.110708 (PMC12550783; doi:10.1016/j.jbc.2025.110708)
Supplement: Figure S4 [file mmc4.pdf]

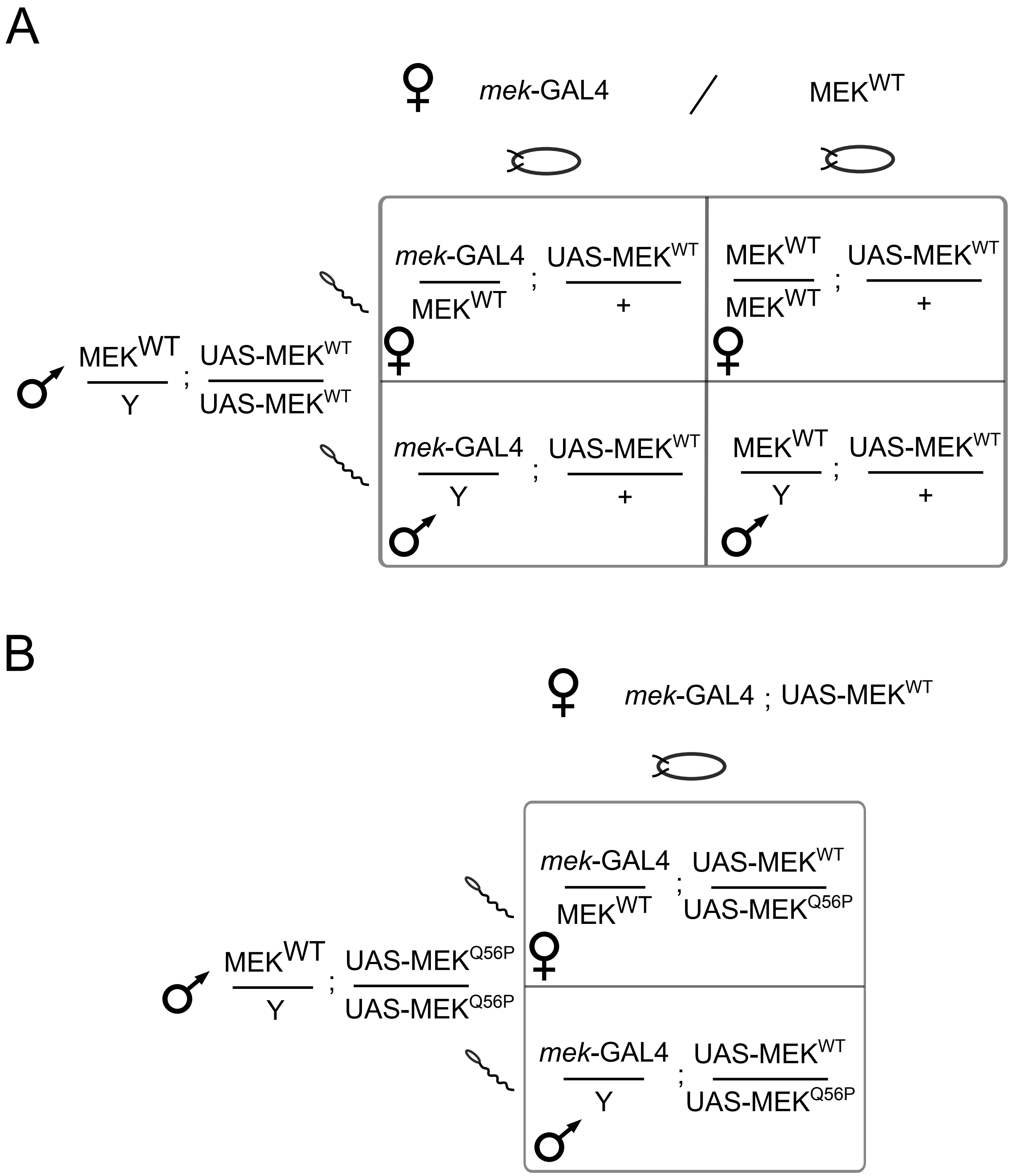

Supplementary Figure S4.

Drosophila genetics of CRIMIC. (A) Hemizygous CRIMIC males or homozygous CRIMIC females are not viable. Female viability is maintained by balancer chromosomes with a wild-type allele of MEK (marked by dominant eye marker, Bar). Punnett square depicts multiple combinations of alleles. CRIMIC males are only viable due to the presence of a rescuing transgene (bottom left square). (B) A homozygous CRIMIC female may be maintained by stably incorporating the rescuing UAS transgene. In this crossing scheme, all progeny inherit the UAS gain-of-function variant of MEK.
