# Supplementary material for: Asymmetric coevolution of the MEK–ERK binding interface
Source: J Biol Chem. 2025 Sep 11;301(10):110708. doi: 10.1016/j.jbc.2025.110708 (PMC12550783; doi:10.1016/j.jbc.2025.110708)
Supplement: Figure S2 [file mmc2.pdf]

```
P39745_Cele     MPTWIPNNLCAQPTTRNAKPPSNGHPQATQQQSAPGSLAYRNSSNIPNGATNHVRQQKWQ
P40417_Dmel     MEEFNSSGSVVNGTGSTEVPQSNA------------------------------------
Q7ZW72_Drer     MATAAVSAPAGGGPNPGSGA----------------------------------------
P28482_Hsap     ----MAAAAAAG-----AGP----------------------------------------
                : .   .  .....   . : ..

P39745_Cele     YTRSGHRKMADGEAVISTVNNVEEVHGQLFEVAPRYVNLSYIGEGAYGMVASALDTITRD
P40417_Dmel     ----------------------EVIRGQIFEVGPRYIKLAYIGEGAYGMVVSADDTLTNQ
Q7ZW72_Drer     ----------------------EMVRGQAFDVGPRYSNLSYIGGGAYGMVCSAYDRDNKV
P28482_Hsap     ----------------------EMVRGQVFDVGPRYTNLSYIGEGAYGMVCSAYDNVNKV
                                      *.::**.*:*:*** :*:***:******.** *. :: 

P39745_Cele     RVAIKKISPFEHQTFCQRTLREIKILNRFKHENIINIQEIIRSETVDSLKDIYIVQCLME
P40417_Dmel     RVAIKKISPFEHQTYCQRTLREITILTRFKHENIIDIRDILRVDSIDQMRDVYIVQCLME
Q7ZW72_Drer     RVAIKKISPFEHQTYCQRTLREIKILLRFKHENIIGINDIIRTPTIDQMKDVYIVQDLME
P28482_Hsap     RVAIKKISPFEHQTYCQRTLREIKILLRFRHENIIGINDIIRAPTIEQMKDVYIVQDLME
                **************:********:** **:*****.*.:*:* .::::::*:****.***

P39745_Cele     TDLYKLLKTQKLSNDHVCYFLYQILRGLKYIHSANVLHRDLKPSNLLLNTTCDLKICDFG
P40417_Dmel     TDLYKLLKTQRLSNDHICYFLYQILRGLKYIHSANVLHRDLKPSNLLLNKTCDLKICDFG
Q7ZW72_Drer     TDLYKLLKTQHLSNDHICYFLYQILRGLKYIHSANVLHRDLKPSNLLLNTTCDLKICDFG
P28482_Hsap     TDLYKLLKTQHLSNDHICYFLYQILRGLKYIHSANVLHRDLKPSNLLLNTTCDLKICDFG
                **********.*****:********************************:**********

P39745_Cele     LARVTDPQTDHTGFLTEYVATRWYRAPEIMLNSKGYTKSIDVWSVGCILAEMLSNRPLFP
P40417_Dmel     LARIADPEHDHTGFLTEYVATRWYRAPEIMLNSKGYTKSIDIWSVGCILAEMLSNRPIFP
Q7ZW72_Drer     LARVADPDHDHTGFLTEYVATRWYRAPEIMLNSKGYTKSIDIWSVGCILAEMLSNRPIFP
P28482_Hsap     LARVADPDHDHTGFLTEYVATRWYRAPEIMLNSKGYTKSIDIWSVGCILAEMLSNRPIFP
                ***::**::********************************:***************:**

P39745_Cele     GKHYLDQLNLILAVVGSPSNADLQCIINDKARSYLISLPHKPKQPWARLYPGADPRALDL
P40417_Dmel     GKHYLDQLNHILGVLGSPSRDDLECIINEKARNYLESLPFKPNVPWAKLFPNADALALDL
Q7ZW72_Drer     GKHYLDQLNHILGILGSPSQEDLNCIINIKARNYLLSLPLRSKVPWNRLFPNADPKALDL
P28482_Hsap     GKHYLDQLNHILGILGSPSQEDLNCIINLKARNYLLSLPHKNKVPWNRLFPNADSKALDL
                *********:**:::****..**.**** ***:** *** :.:.**.:*:*:**..****

P39745_Cele     LDKMLTFNPHNRIDIEQALAHPYLEQYYDPGDEPVCEEPFTLEMEFDDLPKEKLKELIWE
P40417_Dmel     LGKMLTFNPHKRIPVEEALAHPYLEQYYDPGDEPVAEVPFRINMENDDISRDALKSLIFE
Q7ZW72_Drer     LDKMLTFNPHKRIEVEEALAHPYLEQYYDPTDEPVAEAPFKFDMELDDLPKETLKELIFE
P28482_Hsap     LDKMLTFNPHKRIEVEQALAHPYLEQYYDPSDEPIAEAPFKFDMELDDLPKEKLKELIFE
                *:********:**.:*:*************.***::* **..:** **::::.**:**:*

P39745_Cele     EAEAHHRRMEAEAAARN---NGGQNPV
P40417_Dmel     E------------TLKFKERQPDNAP-
Q7ZW72_Drer     E------------TARF---QPGYRP-
P28482_Hsap     E------------TARF---QPGYRS-
                *            ::::   :::..:
```

Supplementary Figure 2.
Multiple sequence alignment of ERK's four reference species: Homo sapiens, Danio rerio, Caenorhabditis elegans, and Drosophila melanogaster.
